# Antioxidant and immune responses of broiler chickens supplemented with *Rhazya stricta* extract in drinking water

**DOI:** 10.14202/vetworld.2021.1437-1449

**Published:** 2021-06-04

**Authors:** Saleh M. Albarrak

**Affiliations:** Department of Veterinary Medicine, College of Agriculture and Veterinary Medicine, Qassim University, Buraydah, Saudi Arabia

**Keywords:** antioxidants, chickens, immunomodulation, *Rhazya stricta*

## Abstract

**Background and Aim:**

*Rhazya stricta* is a herbal plant widely used in traditional medicine due to its proficiency and naturalness with few side effects. In this study, we investigated the impact of using an *R. stricta* extract supplement on broiler chickens’ performance, especially the immune system.

**Materials and Methods:**

In addition to the control group, one group received the methanol extract of *R. stricta* in drinking water for the first 2 weeks before being challenged with sheep erythrocytes (SRBCs), while the other group was challenged with SRBCs without receiving the *R. stricta* treatment. We evaluated cellular immunity by determining the phagocytic activity and lymphocyte (L) proliferation and assessed humoral immunity by quantification of the serum total IgM and IgG. We measured the serum levels of antioxidant enzymes and performed a histological examination of the spleen and the bursa of Fabricius (BF).

**Results:**

Our results indicate a significant enhancement in cellular immunity in the group supplemented with *R. stricta* as demonstrated by a significant increase in the phagocytic activity, L proliferation, and percentages of circulating L (p<0.05). The chickens treated with *R. stricta* exhibit an enhanced humoral response shown by a significant elevation in the serum levels of the total antibodies of the IgM and IgG isotypes, along with a notable increase in BF activity. Furthermore, *R. stricta* supplementation is associated with a significant increase in the serum levels of catalase and superoxide dismutase (p<0.05), along with a significant improvement in broilers’ general performance, body weight, and feed efficiency.

**Conclusion:**

Our results suggest an immunomodulatory effect for the methanol extract of *R. stricta* and highlight the potential use of this plant in preventive and therapeutic medicine.

## Introduction

The utilization of conventional medicine is the backbone of essential medicinal services, especially in developing nations. The use of natural medication in the developed world also is increasing. More people are turning toward traditional medicine, which they believe has fewer harmful and adverse reactions than do Western medications. Beneficial (medicinal) plants remain the source of life-sparing remedies for the majority of people worldwide.

Bioactive constituents derived from plants are utilized as nourishment-added substances, colors, insect repellents, beautifying agents, and perfumes. These compounds are collectively known as secondary metabolites. About 80% of the human population depends on medicinal plants to maintain their well-being and to treat sickness. With the increasing interest in medicinal plants as a source of medication, examining new plants with known therapeutic properties used either in modern or traditional medicine is necessary [1].

Remedial plants are widely utilized in Saudi Arabia because of their natural proficiency without side effects. Indeed, even some herbal plants, including *Rhazya stricta*, have for many years been used to regulate immune responses, with their mechanisms of action still unknown. *R. stricta*, locally called *Harmal*, belongs to the Apocynaceae family and is broadly distributed in the Middle East and the northwestern part of the Indian sub-continent [2]. *R. stricta* is a significant therapeutic plant in Saudi Arabia, growing in the desert regions of the Arabian Peninsula [3].

*R. stricta* is broadly used in traditional medicine to treat illness [4,5], as it is a rich source of terpenoid indole alkaloids and contains glycosides, alkaloids, flavonoids, tannins, and triterpenes [6]. *R. stricta* has been used to relieve malignant growths (cancer) [7], diabetes [8], irritation, and infections [6]. Its more than 100 alkaloids [9] have anti-inflammatory, antidepressant, herbicidal, and antifungal properties [7]. Seed oil from *R. stricta* contains a high amount of d-tocopherol, a significant source of Vitamin E [10].

Recently, a few flavonoid compounds have been derived from *R. stricta* gathered from various regions of Saudi Arabia [4]. The biological activities of *R. stricta* are due to the alkaloids rhazimine, strictanol, sewarine, and tetrahydrosecaminediol, which have numerous capacities as antioxidants, antifungals, and antimicrobials and can affect glucose homeo-balance, blood pressure, the nervous system, and arachidonic acid metabolism [9]. In addition, *R. stricta* can be used as an antihelminthic and anti-inflammatory agent and to treat chronic rheumatism and sore throat [11]. In mice, the alkaloid extracts of *R. stricta* have been shown to improve liver function and protect liver tissue from paracetamol [12]. *R. stricta* also is widely used to treat diabetes either alone or as an adjuvant medication [13]. One study has found that *R. stricta* induces the killing of breast cancer cells through apoptosis [14]. Many alkaloids derived from *R. stricta* can suppress the proliferation of different types of tumors *in vivo* and *in vitro* [6].

Free radicals can decrease the antioxidants of the immune system and alter gene expression, which may lead to the production of abnormal proteins. Antioxidant agents are used to counterbalance the effects of free radicals, protecting against degenerative illnesses. Antioxidant agents are grouped into two major classifications: Natural and synthetic. Butylated hydroxytoluenes, butylated hydroxyanisole (BHA), tertiary butylated hydroquinone, and gallic acid (GA) esters are examples of synthetic antioxidants. These antioxidants can successfully hinder oxidation; can fill in as chelating specialists (e.g., ethylene diamine tetra-acetic acid [EDTA]); and can bind to metals, decreasing their involvement in the process [15].

Synthetic antioxidants are thought to cause or advance negative effects on human health (e.g., mutagenesis and carcinogenesis) [16]. Replacing manufactured antioxidants with natural agents can protect from free-radical-related disorders [15]. Natural antioxidants can control the production of free radicals and inhibit their actions in the biological systems [17]. These antioxidants contain antioxidative enzymes (e.g., superoxide dismutase [SOD], glutathione peroxidase [GSH-PX], and catalase [CAT]) and also non-enzymatic components (e.g., GSH and nutrients C and E) [18]. Many plant extracts contain antioxidant agents [19].

Arachidonic acid metabolism in human blood is inhibited by rhazimine, an alkaloid extracted from *R. stricta* leaves [20]. The lyophilized extract of *R. stricta* has been shown to have an antispasmodic impact on rodent muscles [21]. In one investigation, the antioxidant effects appeared at higher dosages, diminishing the hepatic and renal levels of GSH and elevating the levels of ascorbic acid, although the levels of lipid peroxidation were decreased [22]. Another study examining biochemical parameters in rats, such as liver enzymes, blood lipid profile, and kidney functions [23], found that the fluid extract of *R. stricta* and indole alkaloids significantly increased the levels of serum adiponectin and significantly increased insulin resistance [24].

Multiple studies have demonstrated that the alkaloid extract of *R. stricta* leaves restrained multiplication, colonization, and development of different cancer cell lines (e.g., colon and lung cancers) [25]. *R. stricta* has for some time been utilized in regulating immune responses, with its mechanism of action still unknown. Immunomodulation includes the ability to promote or suppress the immune system [26].

To the best of our knowledge, no study has examined the effects of *R. stricta* supplementation on the performance of the immune system in poultry (chicken). Therefore, this study investigated the humoral and cellular immune responses in broiler (Ross) chickens supplemented with the methanol extract of *R. stricta* in drinking water for the first 2 weeks of life. In addition, we examined the effects of the extract on body weight, daily weight gain, daily feed consumption, and feed conversion ratio. To complete the assessment, we performed a leukogram and histological examination of the spleen and bursa of Fabricius (BF), as well as measured the serum levels of antioxidative enzymes, such as SOD, CAT, and GSH-PX. We evaluated the indicator parameters from Day 1 to Day 42 in a well-controlled experiment done in the chicken housings at the Agricultural and Veterinary Research Station at Qassim University.

## Materials and Methods

### Ethical approval

All the procedures done on broilers were permitted by Qassim University, Saudi Arabia (No. 2332).

### Study period and location

The study was conducted from 10-10-2019 to 21-11-2019 (six weeks) in the chicken housing facility at the Agricultural and Veterinary Research Station of Qassim University.

### Plant extract

*R. stricta* plants were collected from their original territories by Fischer S., #97, in Saudi Arabia from the herbarium collection of Martius during blooming season from winter to spring. The aerial plant pieces were washed with tap water, air-dried, crushed, and then extracted. A total of 200 g of the powdered plant was mixed with 2 L of methanol (99.9%), agitated and left for 72 h, and filtrated; the filtrate was placed in clean containers. This process was repeated multiple times until clear filtrate was acquired. Under reduced pressure using a rotary evaporator (BUTCHI, Switzerland), the methanol extracts were concentrated at a temperature not exceeding 50°C, and the yield rates were recorded. The concentrated extract was stored at 4°C until use.

### Extract preparation

A total of 20 mg of the plant powder was resuspended with 100 mL of methanol (99.9%) and left for 24 h. Using Whatman No. 1 filter paper, the methanol extract was then purified, and the residue was removed. Sodium sulfate was used for dehydration to remove the traces of moisture [27].

### Gas chromatography−mass spectral analysis (GC−MS)

Analysis of the methanol extracts using GC−MS was done following a method described by Soumya *et al*. [27] utilizing Agilent GC (Model 6890N coupled to 5973 Mass Selective Detector, USA). To confirm the presence of phytochemicals, the results were compared with an in-built main library (NIST08.L). GC−MS was fortified with Elite-5 ms (5% diphenyl/95% dimethylpolysiloxane), 30 mm×0.25 mm × 0.25 mm df.

For GC−MS detection, the electron ionization energy of 70eV was used with absolute helium gas as the carrier at a fixed current rate of 1 mL/min. An injection capacity of 2 μl was engaged (split ratio 10:1) with an automatic temperature of 250°C and ion source temperature maintained at 200°C. The oven temperature was modified from 110°C (isothermal for 2 min), with an increase of 10°C/min, to 200°C, then 5°C/min to 280°C for a 9-min isothermal time. Mass spectra were taken at 70 eV, with a scan interval of 0.5 s and pieces from 45 to 450 Da. The complete GC−MS running time was 36 min. The relative % of every integral was calculated by contrasting its average top area with the total areas with software modified to deal with mass spectra and chromatograms.

### Total phenolic content (TPC)

A gram of dried sample was reconstituted with 25 mL of methanol (70%) (v/v). The suspensions were agitated actively in a shady container for 100 min at 100 rpm, followed by centrifugation for 10 min at 3225 g. The supernatant was collected, and the pellet was re-extracted doubly with 15 mL of methanol (70%) for determination of the TPC and antioxidant activity. The extract was saved in the dark at –20°C, and analysis was done within 48 h to avoid oxidation. The TPC was measured according to the Folin−Ciocalteu spectrophotometric analysis; the results were linked to a standard curve of previously organized GA solution [28]. TPC was shown as mg of GA equivalents (GAEs) per gram of dried powder (mg of GAE g^−1^ dw).

### 1,1-dipheny1-2-picrylhydrazyl (DPPH) scavenging activity assay (determination of antioxidant activity)

The DPPH reagent DPPH was utilized to determine the fundamental scavenging activity for the dried sample extract using a method modified by Lu *et al*. [28]. A total of 0.1 mL of the sample was mixed with 2.9 mL of 6×10^−5^ mol of DPPH (methanolic solution). The mixture was kept in the dark place for 1 h and read the absorbance at 517 nm. The Trolox calibration curve was constructed as a function of DPPH radical scavenging activity (RSA) and expressed in %. The obtained data were shown as mmol of Trolox equivalent (TE) per gram of sample (mmol TE g^-1^ dw).

### Experimental chickens and housing

One-day-old chicks (Ross broilers; n=90) were provided by the Al Watania Poultry Company (Al-Qassim, Saudi Arabia). Each chick’s average weight was calculated by dividing the total weight of all the chicks by their total number. Chicks were housed in suitable housing rooms at the Agricultural and Veterinary Experiment Station, Qassim University, Saudi Arabia, in optimal rearing conditions. Feed and water were provided as desired, and the housing facility had a Dicam FSC 2.2 m master unit (Farm Energy and Control Services Lid “Farmex,” Pinewood, Reading RG 303VR, UK) for controlling the ventilation and temperature in each room.

### Chicken feed

Two different feeds were provided by the Al Watania Poultry Company, which included broiler starter and finisher rations formulated to provide the recommended nutritional requirements for broilers according to the National Research Council (Table-1) [29]. The starter feed was given to the chicks from age Day 1 to Week 4, when it was replaced with the finishing feed until the end of the experiment. The feed was soybeans and corn free of antibiotics or growth hormones.

**Table-1:** Proximat analysis of crude protein, crude fat, and crude fiber percentages of the starter mash and crumbs diets.

Calculated analysis	

Starter	Grower-Finisher
Crude Protein (Min) 21.5%	Crude Protein (Min) 18.5%
Crude Fat (Min) 2.5%	Crude Fat (Min) 3.0%
Crude Fiber (Max) 3.5%	Crude Fiber (Max) 3.5%

### Experimental design

The chicks were randomly divided into three groups of 30 birds each. The first group was fed the starter and finisher rations (the control group). The second group was fed the starter and finisher rations and challenged with sheep erythrocytes (SRBCs; the SRBC-challenged group). The third group was given the starter and finisher rations plus the SRBC and the *R. stricta* concentrated extract in the water (only for the first 2 weeks of life; considered the treatment group, the SRBC+*R. stricta* group). Considering the rate of daily water consumption, the recommended dose of the extract was 18 mg/chick [30]. SRBCs were used as a foreign antigen to quantify the humoral response.

A total of 30 mL of whole blood from a healthy young ewe was collected into sterile tubes containing equal amounts of Alsever’s solution, 0.1 molar pH 6.1. The blood samples were centrifuged at 900 g for 10 min to pellet the blood cells. The cells were washed 3 times with phosphate-buffered saline (PBS) (pH 8) and suspended in PBS to 1% concentration to be injected in the chicks [31]. The SRBC-challenged group was injected intramuscularly with 0.5 mL of 1% SRBC at Days 14 and 28.

### Broiler performance

Multiple parameters were used to assess broiler performance, including body weight, weekly weight gain, daily feed consumption, and feed conversion ratio. Chicks in each group were weighed totally and weekly until the end of the experiment at 6 weeks of age. The feed given to each group was recorded daily with an automatic weighing device. Feed residues were weighed at the end of each week. Therefore, feed consumption was noted on a weekly basis and calculated as feed consumed per week over 6 weeks. The feed conversion ratios were then calculated for weeks 2, 3, 4, 5, and 6.

### Blood sampling

Blood samples were collected from at least 3 birds/group on days 14, 28, and 42 through the jugular vein into plain tubes. The blood samples were centrifuged at 900 g for 10 min and left to clot for overnight at 4°C. The serum was aspirated and preserved in sterile tubes at –20°C until use.

### Serum levels of SOD, CAT, and GSH-PX

The antioxidant activity was determined by measurement of GSH-PX, SOD, and CAT (Biodiagnostic Kits, CAT. Nos. 2579, 2563, and 2511, respectively).

### *In vitro* carbon clearance assay

Phagocytic activity was evaluated using the *in vitro* carbon clearance assay [32]. Blood was collected on days 14, 28, and 42 in tubes containing an anticoagulant (heparin). A total of 6 mL of India ink (Pelikan AG D-3000, Hanover, Germany) was added to each blood sample (6 mL ink/1.5 mL of blood). The mixture sample was split into three aliquots containing 0.5 mL each to be incubated at 37°C for 20 and 40 min. Following incubation, 150 mL of each mixture was diluted with 2 mL of saline and centrifuged at 50 g for 4 min. The supernatants were collected and measured by a spectrophotometer at 535 nm, with the background used as 0. The data of the optical density were transformed to a log_2_ scale, and the phagocytic index was calculated as the negative of the slope of the regression of optical density (log_2_) on time (h).

### Glucose consumption assay

Autoclaved red blood cell (RBC) lysis buffer composed of NH4Cl (0.155M) 90 g, KHCO_3_ (0.01M) 10 g, and EDTA (0.1 mM) 370 mg dissolved in 1 L of ddH2O was used to remove or lyse the RBCs to isolate leukocytes. Lysis buffer was diluted 1:10 in ddH2O and passed through a 0.22-mm filter before use. Whole blood (200 mL) was mixed with 2 mL of lysis buffer, incubated at room temperature (28-30°C) for 5 min, and centrifuged at 300× g to remove the lysis buffer.

Lymphocyte (L) proliferation was performed using the glucose consumption test as reported by Kosti *et al*. [33]. Phytohemagglutinin (PHA), a T cell mitogen, was used in this assay. Using a 24-well plate, cells were seeded in triplicate in the presence or absence of 5 μg/mL PHA. Each well had 200 µL of culture medium (RPMI Cat# R6504, Sigma-Aldrich, St. Louis, MO, USA) containing 2×10^6^ cells. The plate was incubated at 37°C in a humidified 5% CO_2_ incubator for 72 h. Using assay kits (Human GmbH, 65205 Wiesbaden, Germany) glucose levels were quantified in the medium by observing the variation in the optical density at 500 nm. L proliferation was measured as the amount of the consumed glucose (mg/dL) minus the glucose level in the non-stimulated cell culture.

### Antibody quantification

Total antibody titer for the IgM and IgG isotypes was determined using commercial isotype immunoglobulin (Ig), chicken-specific Sandwich ELISA Kits according to the manufacturer’s specifications (SunLong Biotech Co. Ltd., HangZhou, China).

### Leukogram

N%, L%, and N: L ratios were counted by the cross-sectional method described by Jain [34]. The number of leucocytes counted/slide was 100, and the counted cells were presented as %.

### Relative weights and histopathology of lymphoid organs (BF and spleen)

A total of three birds from each group were euthanized on days 14, 28, and 42. Total body weights were determined; the birds were opened, and the BF and spleen were carefully removed and weighed separately. The relative weights of the different organs were calculated as % of live body weight.

The collected BF and spleen tissue specimens were immediately fixed in 10% neutral buffered formalin and embedded in paraffin wax. Thin sections of 4-5 mm in thickness were then processed and stained with hematoxylin and eosin (H and E) to examine the changes microscopically according to a method previously described [35].

### Statistical analysis

We assigned the obtained results for statistical analysis using GraphPad Prism version 5 software (GraphPad Software, San Diego, CA, USA). We used analysis of variance to detect significant variations among the groups. For significant differences, we performed the Student−Newman−Keuls test. We recorded all data separately.

## Results

### Chemical composition of *R. stricta*

**Figure-1:**
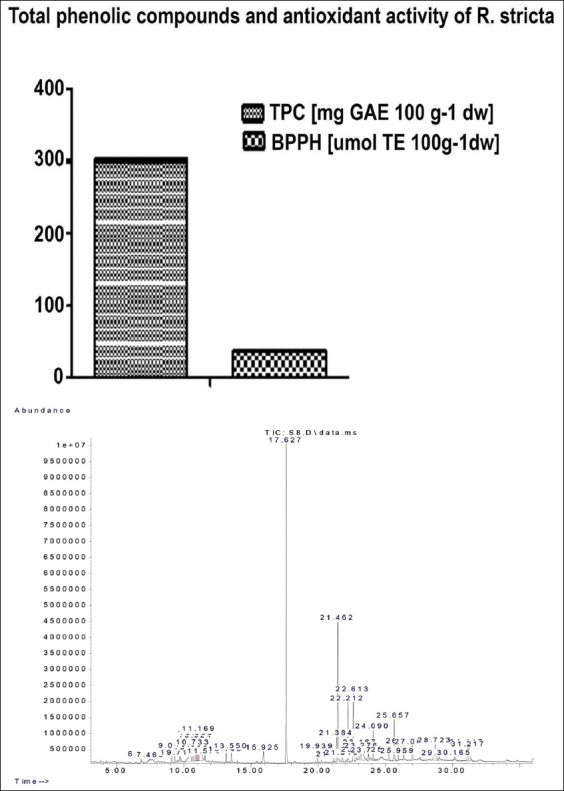
Total phenolic compounds and antioxidant activity of *Rhazya stricta* (A) chromatogram of the methanolic extract of *R. stricta* (B). dw: Dry weight, *: DPPH radical scavenging activity (BPPH-RSA).

We detected signal peaks associated with segregated constituents by GC−MS in the *R. stricta* extract (Figure-1B). Composites greater than 1% are listed in Table-2. Aspidospermidine (28%), uleine (11.76%), 2-hexadecanol (9.27%), 3-o-methyl-d-glucose (6.84%), squalene (3.59%), and d-mannose (3.13%) were the major components present in the extract and were considered to be 50% of its composition.

**Table-2:** Compounds detected by area from *Rhazya stricta* extract.

Name	Peak % area	RT
Aspidospermidine	28	17.626
Uleine	11.76	22.21
2-Hexadecanol	9.27	11.575
3-O-Methyl-d-glucose	6.84	11.166
Squalene	3.59	25.654
d-Mannose	3.13	10.829

### TPC and antioxidant activity of *R. stricta*

We spectrophotometrically determined the TPC of the *R. stricta* extract, presenting the data as mg of GAE per g of the dried *R. stricta* sample. As shown in Figure-1A, the amount of TPC present in the extract was high, reaching 301.99 ± 8.79 mg GAE g^−1^ of the sample. We also assessed the antioxidant activities of the extract using the DPPH reagent DPPH to measure the BPPH-RSA of the dried sample. As shown in Figure-1A, our results indicate that the DPPH-RSA for the *R. stricta* extract was 38.05 ± 6.58 mmol of TE g^−1^.

### Body weight gain and feed conversion rate

The body weight gain in the SRBC+*R. stricta* chicks significantly increased (p<0.05) over that of the chicks in the control group. The feed conversion rate tended to be lower in the control group (1.44±0.33) as compared to the SRBC-challenged and SRBC+*R. stricta* groups (1.40±0.39 and 1.32±0.16, respectively) (Table-3).

**Table-3:** Body weight gain, individual feed consumption, and feed conversion of chickens challenged with sheep erythrocytes following pretreatment with the *Rhazya stricta* extract.

Groups	Initial body weight (g)	Final body weight (day 42)	Δ body weight (body gain)	Individual feed consumption	Feed conversion
Control	45.01±2.54	3111±8.22	3066.0±5.76	4405.9±8.97	1.44±0.33
SRBC	45.72±3.11	3191±7.13	3145.4±6.23	4414.9±4.08	1.40±0.39
*Rhazya stricta* + SRBC	44.85±2.09	3417±6.99	3372.2±4.98^a^	4452.9±6.99	1.32±0.16

Mean±Standard error. Values within the same day having the letter ^a^is significantly different from the control value at p<0.05

### Serum antioxidant enzymes

**Figure-2:**
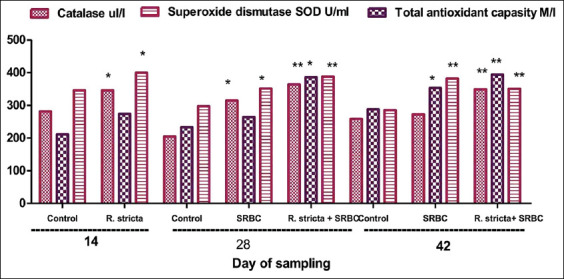
Serum antioxidant enzymes of chickens supplemented with *Rhazya stricta* extract and challenged with sheep erythrocytes. Values within the same day having the marks *,**,*** are significantly different from the control value at p<0.05, p<0.01, and p<0.001, respectively. Mean±Standard error.

We determined the serum antioxidant enzyme activities in the different groups of chicks (Figure-2). The results showed a significant increase in the serum activities of CAT (p<0.01) and SOD (p<0.05) in the SRBC+*R. stricta* chicks. For the SRBC-challenged chicks, serum CAT and SOD activities were significantly elevated (p<0.05) as compared to those in the control chicks.

We noted a significant increase in the serum activities of CAT (p<0.001), total antioxidant capacity (p<0.05), and SOD (p<0.01) on day 28 in the SRBC+*R. stricta* group as compared to the control group. We observed a significant elevation in serum activities of total antioxidant capacity (p<0.05) and SOD (p<0.01) on day 42 in the SRBC-challenged chicks as compared to the control chicks. In addition, we noted a significant increase in serum activities of CAT (p<0.001), total antioxidant capacity (p<0.01), and SOD (p<0.01) in the chicks at the end of the experiment (Day 42) in the SRBC+*R. stricta* and SRBC-challenged groups.

### Phagocytic activities

**Figure-3:**
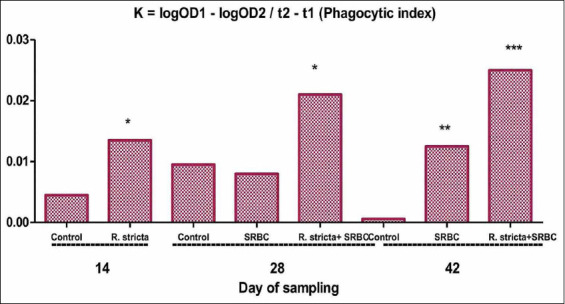
*In vitro* phagocytic index of chickens supplemented with the *Rhazya stricta* extract and challenged with sheep erythrocytes. Values within the same day having the marks *,**,*** are significantly different from the control value at p<0.05, p<0.01, and p<0.001, respectively. Mean±Standard error.

We determined the *in vitro* phagocytic index of chicks on days 14, 28, and 42 following supplementation with the *R. stricta* extract and challenge with SRBCs (Figure-3). The results showed a significant increase in the *in vitro* phagocytic index (p<0.05) of the SRBC+*R. stricta* chicks on day 14 as compared to the control chicks. On day 28, the phagocytic index was significantly elevated (p<0.01) in the SRBC+*R. stricta* group relative to the control group. The index significantly increased on day 42 in the SRBC-challenged group (p<0.01) as well as in the SRBC+*R. stricta* group (p<0.001) as compared to the control group.

### L proliferation determined by glucose consumption

**Figure-4:**
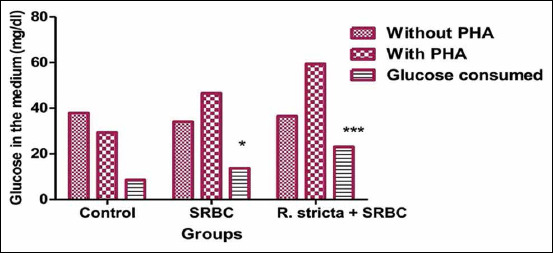
Glucose consumed (mg/dL) by lymphocytes stimulated with phytohemagglutinin of chickens pretreated with *Rhazya stricta* and challenged with sheep erythrocytes. Values within the same day having the marks *,**,*** are significantly different from the control value at p<0.05, p<0.01, and p<0.001, respectively. Mean±Standard error.

As shown in Figure-4, our results showed that the amount of glucose consumed by Ls was significantly higher in the SRBC-challenged chicks (p<0.05) as compared to the control chicks. Similarly, Ls obtained from SRBC+*R. stricta* chicks consumed a significantly higher amount of glucose as compared to Ls in the control chicks (p<0.001).

### Leukogram

**Figure-5:**
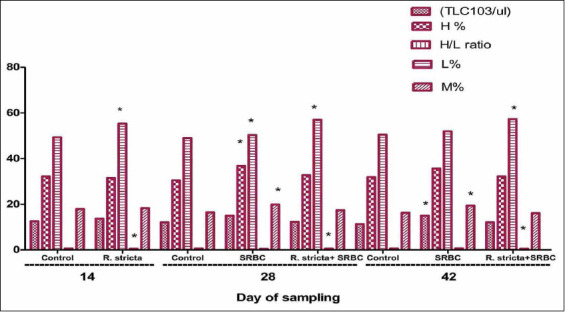
Total leukocytes count (10^3^/mm³); heterophils (H%); lymphocyte (L %), monocyte (M%) and H/L ratio of chickens challenged with sheep erythrocytes post-treatment with *Rhazya stricta*. Values within the same day having the marks *,**,*** are significantly different from control value at p<0.05, p<0.01, and p<0.001, respectively. Mean±Standard error.

Our results revealed a significantly higher L% (p<0.05) and lower H/L (p<0.05) on day 14 in SRBC+*R. stricta* chicks relative to the control chicks. However, we noted similar observations on days 28 and 42. We observed a significant increase in L%, H%, and M% (p<0.05) on day 28 and a significant increase (p<0.05) in TLC and M% in the SRBC-challenged chicks as compared to the control chicks (Figure-5).

### Total IgM and total IgG titers

**Figure-6:**
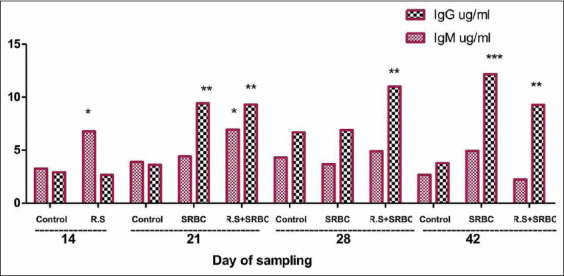
IgG (ug/mL) and IgM (ug/mL) of chickens challenged with sheep erythrocytes post-treatment with the *Rhazya stricta* extract. Values within the same day having the marks *,**,***are significantly different from the control value at p<0.05, p<0.01, and p<0.001, respectively. Mean±Standard error.

We determined the titers of the total IgG and IgM at different times during the experiment using the ELISA technique (Figure-6). On day 14, we observed a significant increase in the concentrations of total IgM in the SRBC+*R. stricta* group as compared to the control group (p<0.05). After the challenge with SRBC, the concentrations of total IgG significantly increased on day 21 as compared to the control group (p<0.01), with the IgM concentrations remaining significantly elevated only in the SRBC+*R. stricta* group (p<0.05). The levels of total IgG were significantly higher in the SRBC+*R. stricta* group (p<0.01) on day 28 and in both the SRBC-challenged (p<0.001) and the SRBC+*R. stricta* (p<0.01) groups on day 42 as compared to the control group.

### Relative weights and histology of BF and spleen

**Figure-7:**
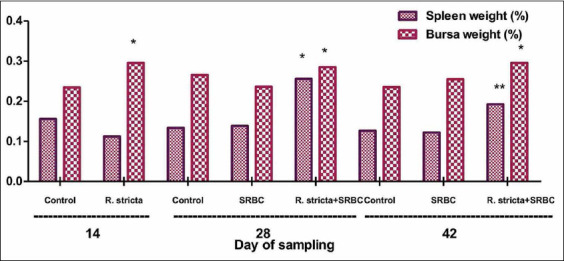
Relative weights of spleen and bursa chickens challenged with sheep erythrocytes following pretreatment with *Rhazya stricta*. Values within the same day having the marks *,**,*** are significantly different from the control value at p<0.05, p<0.01, and p<0.001, respectively. Mean±Standard error.

The BF weights in the SRBC+*R. stricta* chicks were significantly increased (p<0.05) on day 14 as compared to those in the control chicks. On day 28, the weights of both spleen (p<0.05) and BF (p<0.05) were significantly higher in the SRBC+*R. stricta* group as compared to those of the control group. At the end of the experiment (day 42), our data showed a significant increase in the weights of both spleen (p<0.01) and BF (p<0.05) in each of the SRBC+*R. stricta* and SRBC-challenged groups relative to the control group (Figure-7).

**Figure-8:**
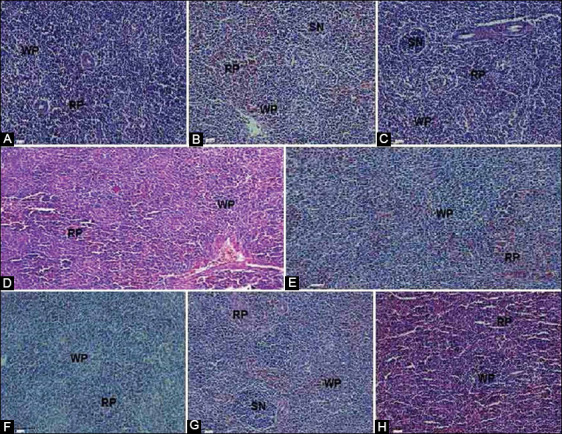
Histological structure of spleen of a control chicken on days 14 (A), 28 (B), and 42 (C), challenged with SRBC on days 28 (D), and 42 (E) and challenged with SRBC after supplementation with the *Rhazya stricta* extract on days 14 (F), 28 (G), and 42 (H). (Hematoxylin and eosin 20×). RP: Red pulp, WP: White pulp, SN: Splenic nodule, SRBC: Sheep erythrocytes.

In the control group, the histological examination of tissue sections of the spleen stained by H and E showed normal histological structures at all ages. As age advanced from day 14 to day 42, different types of splenic nodules were easily visible, as were different types of red and white pulp (Figure-8). In the SRBC-challenged chicks, histological examination of the spleen revealed that the white pulp increased and was easily seen on day 42 as compared to day 28. We noticed that the white pulp increased and the splenic nodules were easily visible in the SRBC+*R. stricta* chicks as they aged. In addition, the red and white pulp were easily demarcated (Figure-8).

The histological examination of the BF in the control chicks on days 14, 28, and 42 stained by H and E showed numerous lymphoid follicles on day 14. The follicles are comprised of the cortex and the medulla. The corticomedullary border, which separates the medulla from the cortex, contained undifferentiated cells and blood capillaries. The connective tissue and blood capillaries were present in the cortex but not in the medulla. A smooth muscular layer rests under the mucosa enclosed by the T. serosa.

**Figure-9:**
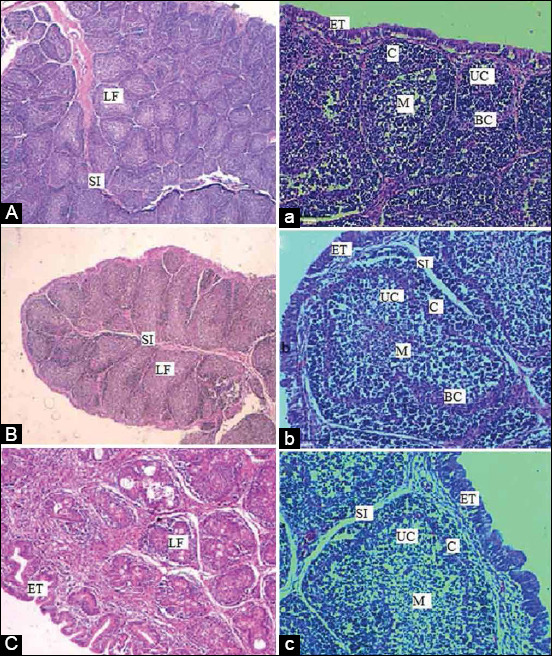
Histological structure of BF of a control chicken at three different ages (14, 28, and 42) with two magnifications. (A, B, C 4×) and (a, b, c 20×). SI: Space interfollicular, C: Cortex, M: Medulla, LF: Lymphoid follicle, ET: Epithelial tissues, UC: Undifferentiated cells, BC: Blood capillaries; BF: Bursa of fabricius.

**Figure-10:**
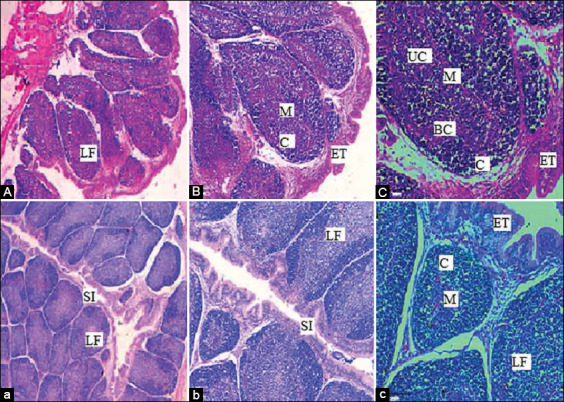
Hematoxylin-eosin staining for BF of a control chicken challenged with two doses of sheep erythrocytes. A, B, C of Bursa of Fabricius collected at day 28 with three magnifications 4×, 10×, and 20×, respectively. a, b, c of BF collected at day 42 with three magnifications 4×, 10×, and 20×, respectively. SI: Space interfollicular, C: Cortex, M: Medulla, LF: Lymphoid follicle, ET: Epithelial tissues, UC: Undifferentiated cells, BC: Blood capillaries; BF: Bursa of fabricius.

**Figure-11:**
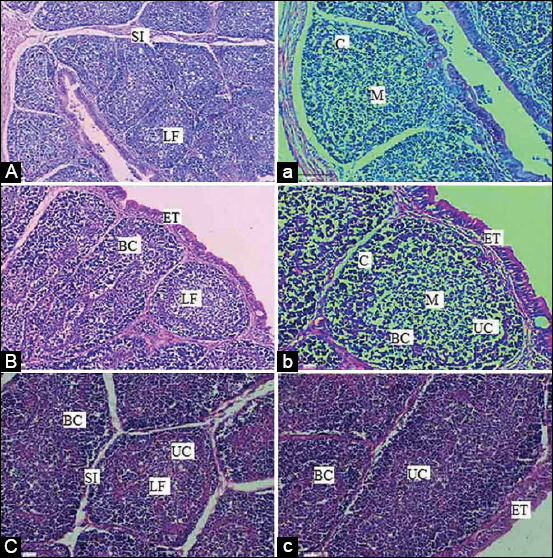
Hematoxylin and eosin staining for BF of chicken supplemented with the *Rhazya stricta* extract before the challenge with two doses of sheep erythrocytes. A, B, C: BF collected on days 14, 28, and 42 with 4×, respectively. a, b, c: BF collected on days 14, 28, and 42 with 20×, respectively. SI: Space interfollicular, C: Cortex, M: Medulla, LF: Lymphoid Follicle, ET: Epithelial tissues, UC: Undifferentiated Cells, BC: Blood capillaries; BF: Bursa of fabricius.

On day 28, BF follicles appeared large and densely populated. The cortex was separated from the medulla by a basal membrane, visible in most sections. On day 42, BF follicles appeared small and densely populated with scattered vacuolation (Figure-9). We noticed that the medulla of the BF of the SRBC-challenged chicks showed a moderate population of Ls, especially on day 42 (Figure-10). The BF of the SRBC+*R. stricta* chicks showed the medulla with an extensive population of Ls, especially on Day 42 (Figure-11).

## Discussion

This study evaluated the humoral and cellular immune responses of broiler chickens following supplementation with the ethanol extract of *R. stricta* in drinking water. The assessment of the humoral immunity included quantification of antibody production as well as histological examination of the BF. We evaluated the effects of the *R. stricta* extract on cellular immunity with leukograms, *in vitro* phagocytic index, glucose consumption tests, and histological examination of the spleen. We also measured the serum levels of the antioxidative enzymes to complete the assessment parallelogram. We recorded measurements of body weight gain, individual feed consumption, and feed conversion.

The TPC of the *R. stricta* extract and their antioxidant activities was 301.99±8.79 and 38.05±6.58, respectively (Figure-1). These values are considered high when compared to other plants, corresponding to a previous work by Iqbal *et al*. [36]. Iqbal *et al*. [36] have reported that the antioxidant capability of the methanolic extracts of *R. stricta* leaves was significantly elevated when compared to the α-tocopherol or the synthetic antioxidant BHA [36]. Another study conducted in Saudi Arabia has demonstrated the high TPC of *R. stricta* extract [4], reporting that the plant exhibited a potent reducing antioxidant ability and free-radical scavenging capacity and recommended this plant for the treatment of certain diseases in humans.

As the chicks advanced in age, the weights of the BF and spleen significantly increased in the SRBC+*R. stricta* group as compared to the control group. Body weight gain significantly increased in this group as well, significantly over that of the control group. The feed conversion rate tended to be lower in the control chicks as compared to the SRBC-challenged or SRBC+*R. stricta* chicks.

No research has investigated the possible role of *R. stricta* as a growth promoter for chickens except for a single study by Al-Homidan *et al*. [37], who have shown that body weight gain and feed efficiency decreased after supplementation of 100 g/kg *R. stricta* in chickens’ diets. The contradictions between our findings and the results of Al-Homidan *et al*. [37] can be explained by the differences in dose, treatment duration, and supplementation type.

In the current study, we evaluated cellular immunity by assessing the *in vitro* phagocytic index, L proliferation, and leukogram. The results of the *in vitro* phagocytic index were significantly increased in the SRBC+*R. stricta* group as compared to the control group on days 14, 28, and 42. Because H% was not affected by the *R. stricta* supplementation, the increase in the phagocytic index in the SRBC+*R. stricta* group is likely due to enhancement in phagocytic activities rather than to an increase in the number of phagocytic cells. The phagocytic index has been shown to be significantly affected by the state of N [38].

Our data show that the amount of glucose consumed by Ls was significantly higher in the chicks challenged with SRBC regardless of *R. stricta* supplementation. However, glucose consumption by Ls of the SRBC+*R. stricta* group was elevated as compared to Ls obtained from the SRBC-challenged group, suggesting that treatment with the *R. stricta* extract slightly enhances L proliferation. The results of the leukogram revealed significantly higher L% and lower H/L ratios on day 14 in the SRBC+*R. stricta* chicks relative to those of the control chicks. However, on days 28 and 42, the results were similar, whereas we observed a significant increase in TLC, H%, and M% in the SRBC-challenged chicks as compared to the control chicks. In addition, the L% significantly increased and H/L% significantly decreased in the SRBC+*R. stricta* chicks relative to the control chicks.

We examined the histology of the spleen to ­complete the assessment of the effects of *R. stricta* supplementation on cellular immunity. As the largest peripheral lymphoid organ, the spleen plays a key role in the immune system responses to bacterial and viral infections. The histological investigation revealed that the broiler spleen is surrounded by a thick splenic capsule and a few trabecula. The red pulp was less distinct and localized within the white pulp, composed mostly of RBCs. Ls of different sizes localized to the white pulp, which contains sheathed arteries and splenic nodules. The histological structures of the spleen in the present investigation correspond to those previously described [39].

In this study, we noted that the white pulp of the spleen increased in the SRBC+*R. stricta* chicks as they advanced in age. As the chicks aged from days 14 to 42, different types of splenic nodules were easily visible. Our findings agree with the previous observations for white leghorn chickens [40].

We assessed the humoral arm of the immune system in the current investigation through the quantification of Ig (Abs) and examined the histological structure of the BF. In birds, the BF is an exceptional organ that grants immunity by providing a microenvironment for the cells of the immune system to develop, mature, and differentiate [41]. The BF contributes to the development of local immune responses by stimulating antibody production through antigens transported to the medulla [42]. However, the complexity of the BF starts at various ages of maturation [43].

The histological examination of the BF of the chicks in the control group revealed the presence of numerous lymphoid follicles on day 14. The corticomedullary border, which contains undifferentiated cells and blood capillaries, divides the follicles into the cortex and medulla as previously described by Aughey and Fyre [43]. The cortex but not the medulla contained the capillary vessels with connective tissues, with the smooth muscular layer located under the mucosa surrounded by the T. serosa.

On day 28, the BF follicles appeared large and densely populated. The cortex was separated from the medulla by a basal membrane, visible in most sections. At the end of the experiment (day 42), the BF follicles appeared small, paler, and less densely populated, with scattered vacuoles. The separation between the cortex and medulla was thickened by connective tissue. At this age, this microscopic appearance is characterized as normal BF, as described by Tarek *et al*. [44]. The BF of SRBC+*R. stricta* chicks showed medullas with an extensive population of Ls, especially on day 42, suggesting that it is still active at this advanced age.

The injection of animals with foreign RBCs is a procedure used to examine the immunomodulatory impact and responses of the immune system without influencing animal well-being [45]. This type of challenge is commonly used in different species, especially to evaluate humoral immunity by determining the increase in antibody titers in response to foreign RBCs [45]. The RBC challenge has been used to quantify IgM and IgG titers in sheep [46], mice [46], and rabbits [47].

We measured Ig at different times using the ELISA technique. On day 14, we noticed a significant increase in the IgM titers of the SRBC+*R. stricta* chicks as compared to the control chicks. The SRBC challenge resulted in a significant increase in the IgG titers, with the IgM titers remaining significantly elevated only in the SRBC+*R. stricta* group on day 21 relative to the control group. We saw a significant increase in the IgG titers in the SRBC+*R. stricta* group on day 28 and in both the SRBC-challenged and SRBC+*R. stricta* groups on day 42 as compared to the control group.

In poultry, the serum levels of IgM and IgG indicate modulation of humoral immunity and stimulation of the adaptive immune responses, as demonstrated by Makkar *et al*. [48]. Chicken IgM resembles its mammalian counterpart structurally and functionally, and it is the dominant isotype generated after the primary exposure to a new antigen [49,50]. Specific IgM responses increase after an initial encounter with an antigen, with responses decreasing following subsequent exposures. The IgM levels were significantly elevated only in the SRBC+*R. stricta* group, suggesting that these chicks may be ready to respond to infections.

The avian IgG is the dominant isotype in the serum and is generated after the IgM isotype in the initial humoral response; IgG is the principal isotype in the subsequent humoral response [51]. The increase in the levels of IgG in the *R. stricta-*supplemented group may suggest a modulation in the humoral arm of the adaptive immune system, where these antibodies effectively contribute to protection against various microbial pathogens [52]. Therefore, biomedicine made from plant extracts may improve essential vital health conditions [53].

## Conclusion

Supplementation of 18 mg/chick from the aerial pieces of *R. stricta* (Harmal) in the drinking water of broiler chickens for 2 weeks shows an enhancement in cell-mediated and humoral immune responses. The results reported in this study highlight the potential use of this plant for therapeutic and preventive purposes. Further studies are needed to isolate its active composites and examine their mode of action against diseases.

## Authors’ Contributions

SMA: Designed and conducted the study, drafted and revised the manuscript, and approved the final manuscript

## References

[1] Dhama K, Karthik K, Khandia R, Munjal A, Tiwari R, Rana R, Khurana S.K, Sana U, Khan R.U, Alagawany M, Farag M.R, Dadar M, Joshi S.K (2018). Medicinal and therapeutic potential of herbs and plant metabolites/extracts countering viral pathogens-current knowledge and future prospects. Curr. Drug Metab.

[2] Gilani S.A, Kikuchi A, Shinwari Z.K, Khattak Z.I, Watanabe K.N (2007). Phytochemical, pharmacological and ethnobotanical studies of *Rhazya stricta* Decne. Phytother. Res.

[3] Adel E.T, Mohamed E.A, Attia A.O, El Dessoky D.S (2012). *In vitro* multiplication of the important medicinal plant, harmal (*Rhazya stricta* Decne). J. Med. Plants Res.

[4] Bukhari N.A, Al-Otaibi R.A, Ibhrahim M.M (2017). Phytochemical and taxonomic evaluation of *Rhazya stricta* in Saudi Arabia. Saudi J. Boil. Sci.

[5] Obaid A.Y, Voleti S, Bora R.S, Hajrah N.H, Omer A.M, Sabir J.S, Saini K.S (2017). Cheminformatics studies to analyze the therapeutic potential of phytochemicals from *Rhazya stricta*. Chem. Cent. J.

[6] Baeshin N.A, Sabir J.S, Qari S.H (2009). Cytogenetic and molecular evaluations of genetic effects of leaf extract of *Rhazya stricta* (Decne) on *Allium cepa*root tip meristems. Egypt. J. Genet. Cytol.

[7] Alagrafi F.S, Alawad A.O, Abutaha N.M, Nasr F.A, Alhazzaa O.A, Alharbi S.N, Alkhrayef M.N, Hammad M, Alhamdan Z.A, Alenazi A.D, Wadaan M.A (2017). *In vitro* induction of human embryonal carcinoma differentiation by a crude extract of *Rhazya stricta*. BMC Complement. Altern. Med.

[8] Ahmed A, Asad M.J, Ahmad M.S, Qureshi R, Shah S.I, Gul H, Gulfraz M (2015). Antidiabetic and hypolipidemic potential of *Rhazya stricta* Decne extract and its fractions. Int. Curr. Pharm. J.

[9] Ahmad A, Wei Y, Ullah S, Shah S.I, Nasir F, Shah A, Iqbal Z, Tahir K, Khan U.A, Yuan Q (2017). Synthesis of phytochemicals-stabilized gold nanoparticles and their biological activities against bacteria and *Leishmania*. Microb. Pathog.

[10] Nehdi I.A, Sbihi H.M, Tan C.P, Al-Resayes S.I (2016). Seed oil from Harmal (*Rhazya stricta* Decne) grown in Riyadh (Saudi Arabia):A potential source of d-tocopherol. J. Saudi Chem. Soc.

[11] Ali B.H, Al-Qarawi A.A, Bashir A.K, Tanira M.O (2000). Phytochemistry, pharmacology and toxicity of *Rhazya stricta* Decne:A review. Phytother. Res.

[12] Ali B.H, Bashir A.K, Rasheed R.A (2001). Effect of the traditional medicinal plants *Rhazya stricta*, *Balanitis aegyptiaca* and *Haplophylum tuberculatum* on paracetamol?induced hepatotoxicity in mice. Phytother. Res.

[13] Al-Hasawi Z.M, Al-Harbi H.A (2014). Effect of *Rhazya stricta* dense leaf extract on the liver and kidney tissue structure of albino mice. Global Adv. Res. J. Environ. Sci. Toxicol.

[14] Bernstein J.A, Liu N, Knorr B.A, Smugar S.S, Hanley W.D, Reiss T.F, Greenberg S (2011). MK-0633, a potent 5-lipoxygenase inhibitor, in chronic obstructive pulmonary disease. Respir. Med.

[15] He L, He T, Farrar S, Ji L, Liu T, Ma X (2017). Antioxidants maintain cellular redox homeostasis by elimination of reactive oxygen species. Cell. Physiol. Biochem.

[16] Xu D.P, Li Y, Meng X, Zhou T, Zhou Y, Zheng J, Zhang J.J, Li H.B (2017). Natural antioxidants in foods and medicinal plants:Extraction, assessment and resources. Int. Mol. Sci.

[17] Maarman G.J (2017). Natural antioxidants as potential therapy, and a promising role for melatonin against pulmonary hypertension. Pulmonary Vasculature Redox Signaling in Health and Disease.

[18] Meghwani H, Prabhakar P, Mohammed S.A, Seth S, Hote M.P, Banerjee S.K, Arava S, Ray R, Maulik S.K (2017). Beneficial effects of aqueous extract of stem bark of *Terminalia arjuna* (Roxb.), An ayurvedic drug in experimental pulmonary hypertension. J. Ethnopharmacol.

[19] Simioni C, Zauli G, Martelli A.M, Vitale M, Sacchetti G, Gonelli A, Neri L.M (2018). Oxidative stress:Role of physical exercise and antioxidant nutraceuticals in adulthood and aging. Oncotarget.

[20] Saeed S.A, Simjee R.U, Mahmood F, Sultana N (1993). Rhazimine from *Rhazya stricta*:A dual inhibitor of arachidonic acid metabolism and platelet-activating factor-induced platelet aggregation. Planta Med.

[21] Tanira M.O.M, Ali B.H, Bashir A.K, Wasfi I.A, Chandranath I (1996). Evaluation of the relaxant activity of some United Arab Emirates plants on intestinal smooth muscle. J. Pharm. Pharmacol.

[22] Ali B.H, Alqarawi A.A, Bashir A.K, Tanira M.O (2000). Antioxidant action of extract of the traditional medicinal plant *Rhazya stricta* Decne. in rats. Phytother. Res.

[23] Baeshen N.A, Lari S.A, Aldoghaither H.A, Elkady A.I (2010a). Biochemical evaluation of the effect of *Rhazya stricta* aqueous leaves extract in liver and kidney functions in Rats. Nat. Sci.

[24] Baeshen N, Lari S, Al Doghaither H.A, Ramadan H.A (2010b). Effect of *Rhazya stricta* extract on rat adiponectin gene and insulin resistance. J. Am. Sci.

[25] Elkady A.I, Hussein R.A, Abu-Zinadah O.A (2014). Differential control of growth, apoptotic activity and gene expression in human colon cancer cells by extracts derived from medicinal herbs, *Rhazya stricta* and *Zingiber officinale* and their combination. World J. Gastroenterol.

[26] Patwardhan B, Kalbag D, Patki P.S, Nagsampagi B.A (1990). Search of immunomodulatory agents:A review. Indian Drugs.

[27] Soumya V, Muzib Y.I, Venkatesh P, Hariprasath K (2014). GC-MS analysis of *Cocos nucifera* flower extract and its effects on heterogeneous symptoms of polycystic ovarian disease in female Wistar rats. Chine. J. Nat. Med.

[28] Lu J, Zhao H, Chen J, Fan W, Dong J, Kong W, Sun J, Cao Y, Cai G (2007). Evolution of phenolic compounds and antioxidant activity during malting. J. Agric. Food Chem.

[29] National Research Council (1994). Nutrient Requirements of Poultry.

[30] Alharbi K.B, Mousa H.M, Ibrahim Z.H, El-Ashmawy I (2017). Hepatoprotective effect of methanolic extracts of *Prosopis farcta* and *Lycium shawii* against carbon tetrachloride-induced hepatotoxicity in rats. J. Biol. Sci.

[31] Trombetta C.M, Ulivieri C, Cox R.J, Remarque E.J, Centi C, Perini D, Piccini G, Rossi S, Marchi S, Montomoli E (2018). Impact of erythrocyte species on assays for influenza serology. J. Prev. Med. Hyg.

[32] Spinu M, Degen A.A (1993). Effect of cold stress on performance and immune responses of Bedouin and White Leghorn hens. Br. Poult. Sci.

[33] Kosti O, Byrne C, Cocilovo C, Willey S.C, Zheng Y.L (2010). Phytohemagglutinin-induced mitotic index in blood lymphocytes:A potential biomarker for breast cancer risk. Breast Cancer.

[34] Jain N.C (1986). Veterinary Hematology.

[35] Guo L, DeRoche T.C, Salih Z.T, Qasem S.A (2018). Routine hematoxylin and eosin stain is specific for the diagnosis of cytomegalovirus infection in gastrointestinal biopsy specimens. Int. J. Surg. Pathol.

[36] Iqbal S, Bhanger M.I, Akhtar M, Anwa F, Ahmed K.R, Anwer T (2006). Antioxidant properties of methanolic extracts from leaves of *Rhazya stricta*. J. Med. Food.

[37] Al-Homidan A, Al-Qarawi A.A, Al-Waily S.A, Adam S.E (2002). Response of broiler chicks to dietary *Rhazya stricta* and Nigella sativa. Br. Poult. Sci.

[38] Dey A, Allen J, Hankey-Giblin P.A (2015). Ontogeny and polarization of macrophages in inflammation:Blood monocytes versus tissue macrophages. Front. Immunol.

[39] Hodges R.D (1974). The Histology of the Fowl.

[40] Khan M.Z, Hashimoto Y, Asaduzzman M (1998). Development of T-cell sub-populations in postnatal chicken lymphoid organs. Vet. Arhiv.

[41] Martelli D, Farmer D.G, Yao S.T (2016). The splanchnic anti-inflammatory pathway:Could it be the efferent arm of the inflammatory reflex?. Exp. Physiol.

[42] Olah I, Glick B (1992). Follicle-associated epithelium and medullary epithelial tissue of the bursa of Fabricius are two different compartments. Anatom. Record.

[43] Aughey E, Frye F.L (2001). Comparative Veterinary Histology with Clinical Correlates.

[44] Tarek K, Mohamed M, Hassina B, Messaouda I (2013). Histological study of the bursa of Fabricius of broiler chickens during heat stress. Int. J. Poult. Sci.

[45] Geng T, Guan X, Smith E.J (2015). Screening for genes involved in antibody response to sheep red blood cells in the chicken, *Gallus gallus*. Poult. Sci.

[46] McAllister E.J, Apgar J.R, Leung C.R, Rickert R.C, Jellusova J (2017). New methods to analyze B cell immune responses to thymus-dependent antigen sheep red blood cells. J. Immunol.

[47] Qiu C.F, Lei L.S, Wu Y.Y, Yu C.L, Zhu Z.G, Chen N.N, Wu S.G (2009). Establishment of mouse model of humoral immune response using rabbit red blood cells as the antigen. J. Southern Med. Univ.

[48] Makkar S, Rath N.C, Packialakshmi B, Huff W.E, Huff G.R (2015). Nutritional effects of egg shell membrane supplements on chicken performance and immunity. Poult. Sci.

[49] Grönwall C, Silverman G.J, Natural I.G.M (2014). beneficial autoantibodies for the control of inflammatory and autoimmune disease. J. Clin. Immunol.

[50] Ehrenstein M.R, Notley C.A (2010). The importance of natural IgM:Scavenger, protector and regulator. Nat. Rev. Immunol.

[51] Aboelsoued D, Abo-Aziza F.A.M, Mahmoud M.H, Abdel Megeed K.N, Abu El Ezz N.M.T, Abu-Salem A.M (2019). Anticryptosporidial effect of pomegranate peels water extract in experimentally infected mice with special reference to some biochemical parameters and antioxidant activity. J. Parasit. Dis.

[52] Jeurissen S.H (1993). The role of various compartments in the chicken spleen during an antigen-specific humoral response. Immunology.

[53] Dhama K, Latheef S.K, Mani S, Abdul Samad H, Karthik K, Tiwari R, Khan R.U, Alagawan M, Farag M.R, Alam G.M, Laudadio V, Tufarelli V (2015). Multiple beneficial applications and modes of action of herbs in poultry health and production-a review. Int. J. Pharmacol.

